# Improving the Quality and Use of Malaria Surveillance Data: Results from Evaluating an Integrated Malaria Information Storage System at the Health Facility Level in Selected Districts in Mozambique

**DOI:** 10.4269/ajtmh.23-0138

**Published:** 2024-01-23

**Authors:** Ann-Sophie Stratil, Neide Canana, Maria Rodrigues, Sarmento Armando, Sergio Gomane, Edson Zandamela, Kevin Baker, Arantxa Roca-Feltrer, Baltazar Candrinho

**Affiliations:** ^1^ Malaria Consortium, London, United Kingdom;; ^2^ Malaria Consortium, Maputo, Mozambique;; ^3^ Department of Global Public Health, Karolinska Institute, Stockholm, Sweden;; ^4^ National Malaria Control Programme at Ministry of Health, Maputo, Mozambique

## Abstract

Mozambique addressed critical malaria surveillance system challenges by rolling out an integrated malaria information storage system (iMISS) at the district level in February 2021. The iMISS integrates malaria data from existing systems across thematic program areas to improve data availability and use. In seven districts, the platform was extended to health facilities (HFs), allowing HFs to access iMISS and use tablets to submit monthly malaria reports to a central database, eliminating the need for paper-based reporting to districts. A structured evaluation of the iMISS rollout to HFs was carried out in February–July 2021. The four evaluation areas were data quality (reporting rate, timeliness, and fidelity) of monthly malaria reports electronically submitted to the iMISS, adoption of the iMISS for data-informed decision-making, system maintenance, and acceptability of the iMISS among target users. All 94 HFs in the seven targeted districts were assessed. Over the 6-month period, 86.1% of reported cases on the iMISS were consistent with cases recorded in paper-based reports, allowing for up to 10% discrepancy. In addition, 69.0% of expected monthly district meetings were held, and information from iMISS was discussed during 58.6% of these meetings. Maintenance issues, mostly related to tablet access and internet connectivity, were experienced by 74.5% of HFs; 33.7% of issues were resolved within 1 month. The iMISS and electronic submission of malaria reports were well accepted by HF- and district-level users. Continued political commitment and timely execution of issue management workflows are crucial to ensure trust in the new platform and facilitate higher levels of data use.

## INTRODUCTION

With an estimated 10 million cases in 2020, Mozambique has one of the highest burdens of malaria in the world and, as such, is a core target for the WHO and RBM Partnership to End Malaria country-led “high burden to high impact” initiative.^1^ Surveillance system assessments carried out in 2016 and later in 2018 identified critical challenges in the malaria surveillance system, namely multiple data sources and systems as well as poor accessibility and integration of data without automated analysis mechanisms.^2^ The malaria surveillance system at the time relied mainly on the Sistema de Informação para Saúde de Monitoria e Avaliação (SISMA), a District Health Information Software 2 (DHIS2)–supported platform deployed at district levels for reporting routine malaria data as part of Mozambique’s integrated health information system. Monthly malaria reports, which are prepared at the health facility (HF) level and contain aggregated malaria case data compiled from HF and community health worker registers, are submitted to district health offices (DHOs) as paper-based reports and subsequently entered into the SISMA. Other key malaria surveillance data were captured in parallel systems, which represented a challenge to the National Malaria Control Programme (NMCP) and its partners in terms of progress monitoring and informed decision-making. Because SISMA alone was not sufficiently meeting information demands, the NMCP recognized the need for a malaria information platform that could integrate malaria information systems and be deployed to all HFs to improve data reporting, accessibility, and use for decision-making.

To achieve this goal, the NMCP prioritized data-driven decision-making and the development of such a platform in its 2017–2022 National Malaria Control Strategic Plan^3^ and, between 2018 and 2020, developed an integrated malaria information storage system (iMISS) in collaboration with implementing partners.^4^ The iMISS, like the SISMA, is a DHIS2-based platform with the following primary objectives: 1) to function as a data warehouse by retrieving and storing malaria data from different routine information systems, while adding electronic reporting through web or mobile interfaces for programmatic areas that have historically relied on paper and ad hoc tools such as spreadsheets and 2) to enable malaria staff to monitor key indicators and provide quality evidence in order to plan and implement timely responses through the configuration of standard indicators and customized data visualizations and outputs. The iMISS is organized into eight thematic modules: Case surveillance, Commodities, Vector control, Entomological surveillance, Communications, Data quality audits, Supervision, and Surveys. Each module receives data inputs from different data systems. The iMISS was to be rolled out in two distinct but overlapping phases. Phase 1 prioritized building the web-based data warehouse within DHIS2 for all existing and essential routine data streams, whereas phase 2 focused on extending the data warehouse and including the configuration and integration of mobile tools to extend reporting to the HF level.^5^

**Figure 1.**
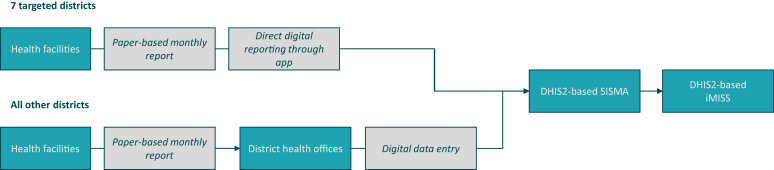
Data flow of monthly malaria report data to the iMISS. DHIS2 = District Health Information Software 2; iMISS = integrated malaria information storage system; SISMA = Sistema de Informação para Saúde de Monitoria e Avaliação.

Phase 1 modules of the iMISS were rolled out in February 2021 and allowed national- and district-level staff to start using the system. Experiences from other countries showed that extending access to an electronic reporting system beyond the district level can increase ownership.^6^ Phase 2 functionalities (i.e., the extension of the iMISS to the HF level) were therefore trialed in seven districts. Selected HF malaria focal points were provided with tablets to allow data access and to facilitate electronic reporting of HF-level monthly malaria reports through an app linked to the SISMA. Data could then flow from the SISMA to the DHIS2-based iMISS. Introducing electronic reporting at the HF level saves the additional step of submitting paper-based reports to DHOs (Figure 1).

This evaluation was designed to assess four dimensions of the new system: data quality of monthly malaria reports electronically submitted to the iMISS, adoption of the iMISS for data-informed decision-making, system maintenance, and acceptability of the iMISS among target users. Lessons learned from this evaluation aim to optimize the scale-up of the iMISS and rollout of electronic reporting to HFs.

## MATERIALS AND METHODS

### Evaluation design and setting.

The evaluation took place from February to July 2021, covering the first 6 months after the rollout of the iMISS at the HF level in February 2021. Prior to the rollout, between October and December 2020, users at national, district, and HF levels were trained on how to use the iMISS and electronically submit data. A technical advisory group consisting of representatives from the national program and implementing partners oversaw the rollout. Regular supervision activities were conducted throughout this period to ensure uptake of the system and timely identification and resolution of operational issues.

**Figure 2.**
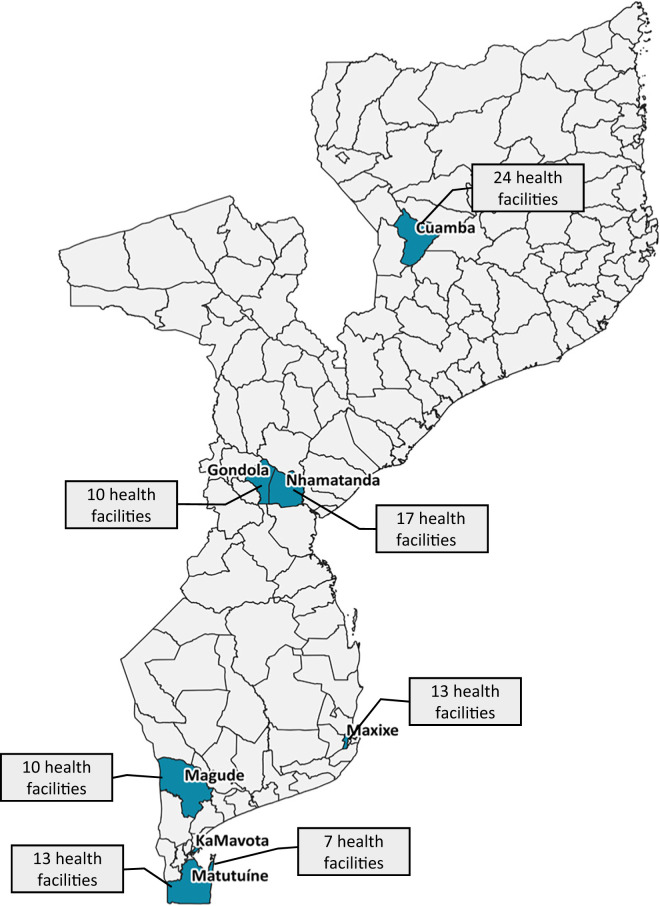
District sample for evaluation.

Data quality was assessed through reporting rate, timeliness, and fidelity of monthly malaria reports electronically submitted on the iMISS. Adoption of the iMISS for data-informed decision-making was assessed through monthly meetings at the district level. System maintenance was assessed in relation to the HF’s ability to access the iMISS platform and digitally submit monthly reports through tablets. Acceptability of the iMISS among key users was assessed through a qualitative thematic framework approach probing the following themes: 1) perceived data quality, 2) workload, 3) ease of iMISS data reporting and dashboard functionalities, 4) usefulness, and 5) operational/technical issues. The evaluation was conducted at the 94 HFs where the iMISS was rolled out. These HFs were located across Mozambique in the districts of Cuamba, Gondola, Nhamatanda, Maxixe, Magude, KaMavota, and Matutuine (Figure 2).

### Sampling and data collection.

Information on data use and system maintenance was collected from malaria focal points at each of the 94 HFs and seven DHOs through monthly questionnaires conducted by telephone. Questionnaires directed at district-level malaria focal points focused on data use during monthly district meetings. These meetings are attended by malaria focal points from the HF level and should include a review of malaria-related information on the iMISS. Questionnaires directed at HF-level malaria focal points focused on maintenance issues related to accessing the iMISS platform and electronic submission of monthly malaria reports, including the type of issue and time to resolution. Data collected during telephone interviews were electronically captured through KoBoToolbox. Information on timely report submission and fidelity of electronically submitted reports was compared against paper-based reports. The data from paper-based reports were obtained from photos of the “original” paper-based monthly malaria reports.

Key informant interviews were conducted in July 2021 with district-level malaria focal points from seven DHOs and a purposive selection of HF malaria focal points from six rural (farthest from the district capital) and six urban (based in or closest to the district capital) facilities. Malaria focal points were selected if they had been working in their function for at least 6 months prior to the interview. Interviews were conducted in Portuguese, audio recorded, and later transcribed.

### Statistical methods and variables.

Data quality was assessed in line with national protocols for data quality assurance. 1) Reporting rate was assessed as the number of electronically submitted reports on the SISMA over the number of expected monthly electronic reports. 2) Timely submission of electronic reports on the SISMA was assessed as the electronically transmitted reports that were submitted between the 21st and the 25th of each month over the number of all monthly electronic reports that were submitted. 3) Fidelity of electronically submitted reports against paper-based reports was assessed as the number of rapid diagnostic test- and microscopy-confirmed malaria cases from electronically submitted reports on the iMISS over the number of malaria cases reported in paper-based reports. Only submitted reports were considered. If the number of malaria cases reported on the iMISS was within ±10% of the cases recorded in paper-based reports, the data were considered “accurate” and otherwise as “inaccurate.” In addition, the mean proportion of paper-based reported malaria cases on the iMISS and mean absolute difference between the number of malaria cases on the iMISS and paper-based reports were assessed. Because the data structure did not allow differentiation between missing data and zero, a conservative approach replaced the missing data with zero. Sensitivity analysis in which records with missing data were removed from the analysis showed similar results in terms of binary fidelity outcome (accurate or inaccurate) but less pronounced discrepancies concerning average absolute differences. Consistency between monthly malaria report data on the SISMA and the iMISS was quantified using the same approach; however, data were classified as completely consistent only if discrepancies were zero. Adoption of the system for data-informed decision-making was measured by the proportion of expected monthly meetings that took place and the proportion of monthly meetings at the district level that took place where iMISS data were discussed. System maintenance was assessed through the proportion of HFs that experienced an issue related to digital data submission and accessing the iMISS platform through tablets at any point and resolution time of issues. Data were analyzed using STATA 13 (Stata Statistical Software: Release 13; StataCorp LP, College Station, TX). Qualitative data were coded in line with the predefined themes described above using Microsoft Excel (Redmond, WA). The analysis was based on Portuguese interview transcripts and later translated to English.

## RESULTS

### Sample.

Monthly data were collected from 94 HFs across seven districts from February to July 2021. Of the 94 HFs, 88% (83/94) were located in rural settings and 12% (11/94) were in urban settings. The HFs recorded an average of 32.5 (SD, 79.1) malaria cases per month over the evaluation period. District-level malaria focal points (*N* = 7) participating in the key informant interviews had been in their role for an average of 1.3 (SD, 0.47) years, and 71% (5/7) had a medium educational level, defined as below a university degree. The HF malaria focal points had been in their role for longer, an average of 3.9 (SD, 1.49) years, and a slightly lower proportion (67% [8/12]) had a medium educational level (Table 1).

**Table 1:** Key characteristics of sample

Variable	Level	Health Facility	District
Number of facilities, districts	Total	94	7
Setting, % (*n*/*N*)	Urban	12% (11/94)	–
	Rural	88% (83/94)	–
Number of selected participants for key informant interviews	Total	12	7
Setting of selected malaria focal points	Rural	50% (6/12)	–
	Urban	50% (6/12)	–
Time in function, years, mean (SD)	–	3.9 (1.49)	1.3 (0.47)
Educational level, % (*n*/*N*)	University	25% (3/12)	29% (2/7)
	Medium	67% (8/12)	71% (5/7)
	Basic	8% (1/12)	0% (0/7)

### Reporting rate and timeliness of electronically submitted monthly malaria reports on the SISMA.

The reporting rate for monthly malaria data that were submitted electronically to the SISMA was 99.3% (560/564), and 99.8% (559/560) of submitted reports were submitted on time. The missing reports (four) were all from the same HF. There were two instances where the same report was submitted twice; these were excluded from the denominator.

### Fidelity of number of malaria cases reported on the iMISS compared with paper-based monthly reports.

**Figure 3.**
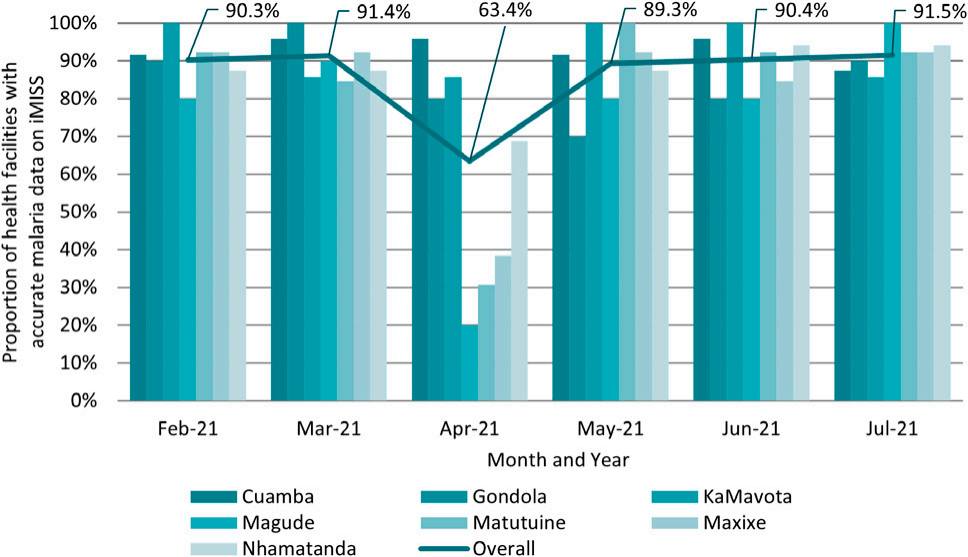
Proportion of health facilities with accurate number of malaria case data on the iMISS compared with monthly paper-based reports by district. iMISS = integrated malaria information storage system.

On the iMISS, 86.1% (482/560) of electronically submitted numbers of malaria cases were within 10% of cases reported through paper-based reports (Table 2). On average, 94.9% (SD, 35.1%) of cases recorded in paper-based reports were captured on the iMISS per HF and month. This translates to a mean average of 25.9 (SD, 221.0) malaria cases per HF per month not captured on the iMISS. Most inconsistencies between the numbers of malaria cases in paper-based reports and those reported on the iMISS were recorded in April, when only 63.4% (59/93) of HFs showed an accurate number of malaria cases on the iMISS (Figure 3). In 73.5% (25/34) of cases, these were due to inconsistencies between the iMISS and the SISMA (see section on discrepancies between malaria cases reported on the SISMA and the iMISS). More than half (51.1% [48/94]) of HFs showed inaccurate malaria cases on the iMISS compared with paper-based reports at some point during the 6-month evaluation period. Overall, 22.3% (21/94) of HFs showed inaccurate malaria case data more than once during the evaluation period.

**Table 2:** Fidelity of number of malaria cases reported on the iMISS compared with monthly paper-based reports

Month	Proportion of HFs with Accurate Malaria Cases on iMISS, % (*n*/*N*)	Average Proportion of Paper-Based Report Cases on iMISS, % (SD)	Average difference between number of malaria cases on iMISS and paper-based reports, mean (SD)
February 2021	90.3% (84/93)	99.1% (18.5%)	−22.1 (246.4)
March 2021	91.4% (85/93)	99.5% (25.4%)	−17.0 (192.6)
April 2021	63.4% (59/93)	68.1% (49.3%)	−84.7 (284.1)
May 2021	89.3% (83/93)	103.6% (34.0%)	−5.6 (230.4)
June 2021	90.4% (85/94)	102.4% (40.7%)	−14.1 (221.7)
July 2021	91.5% (86/94)	96.4% (18.6%)	−12.0 (104.6)
Total	86.1% (482/560)	94.9% (35.1%)	−25.9 (221.0)

HF = health facility; iMISS = integrated malaria information storage system.

### Discrepancies between malaria cases reported on the SISMA and the iMISS.

For 92.1% (516/560) of records, the number of malaria cases was consistent between the SISMA and the iMISS. During the evaluation period from February to July 2021, April showed the highest discrepancies, with only 63.4% (59/93) of HFs reporting the same number of malaria cases as on the SISMA and the iMISS, translating to an average of only 71.5% (SD, 47.5%) of SISMA malaria cases captured on the iMISS that month (Table 3). In the same month, fidelity between the iMISS and monthly reports was at its lowest, with only 63.4% (59/93) of HFs reporting accurate numbers of malaria cases on the iMISS (Table 2). Overall, the iMISS captured an average of 11.5 (SD, 87.6) fewer malaria cases than the SISMA per HF per month.

**Table 3:** Consistency between the numbers of malaria cases reported on the SISMA and iMISS

Month	Proportion of Health Facilities with Complete Consistency between SISMA and iMISS, % (*n/N*)	Average Proportion of Malaria Cases Reported on SISMA on iMISS, % (SD)	Average Difference between Absolute Malaria Cases Reported on SISMA and iMISS, mean (SD)
February 2021	95.7% (89/93)	99.3% (10.7%)	0.1 (0.5)
March 2021	100% (93/93)	100% (0%)	0 (0)
April 2021	63.4% (59/93)	71.5% (47.5%)	−69.6 (186.8)
May 2021	97.8% (91/93)	100.7% (5.2%)	6.9 (65.3)
June 2021	96.8% (91/94)	98.2% (10.0%)	−6.8 (57.3)
July 2021	98.9% (93/94)	100.2% (1.7%)	0 (0.4)
Total	92.1% (516/560)	95.0% (22.9%)	−11.5 (87.6)

iMISS = integrated malaria information storage system; SISMA = Sistema de Informação para Saúde de Monitoria e Avaliação.

### Adoption of the iMISS for data-informed decision-making.

Only 69.0% (29/42) of expected monthly meetings at the district level took place. Gondola (2/6), Cuamba (3/6), and Nhamatanda (3/6) held the fewest meetings. In 58.6% (17/29) of the meetings that took place, information from the iMISS was discussed. KaMavota had the highest use rate at the district level, with the iMISS discussed during all meetings that took place (5/5), whereas Maxixe showed lowest use at the district level (1/5). Discussion topics included information from supervision reports on data quality, vector control, and case management as well as surveillance data regarding monthly malaria trends, number of consultations, and commodities.

### System maintenance.

**Figure 4.**
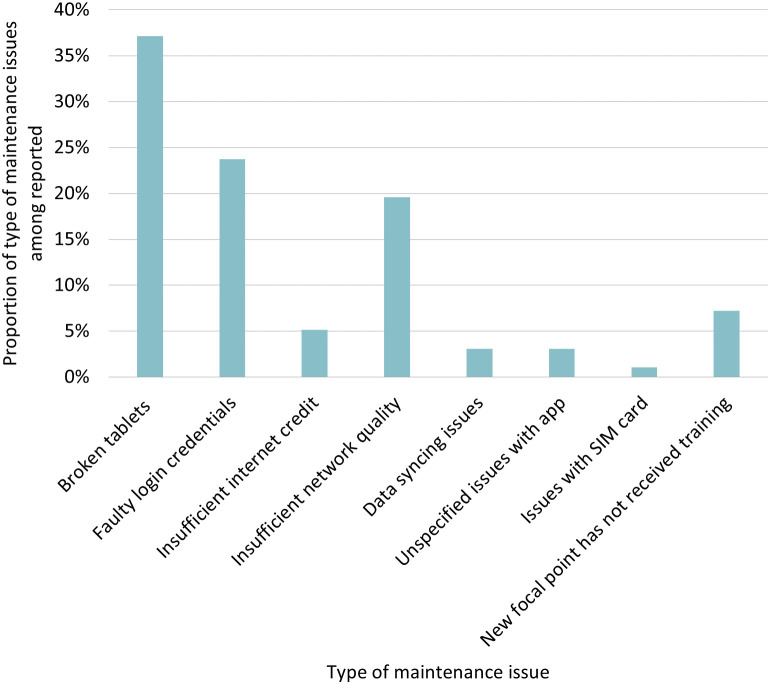
Type of maintenance issues reported with iMISS access and digital data submission at health facilities (*N* = 97). iMISS = integrated malaria information storage system.

**Figure 5.**
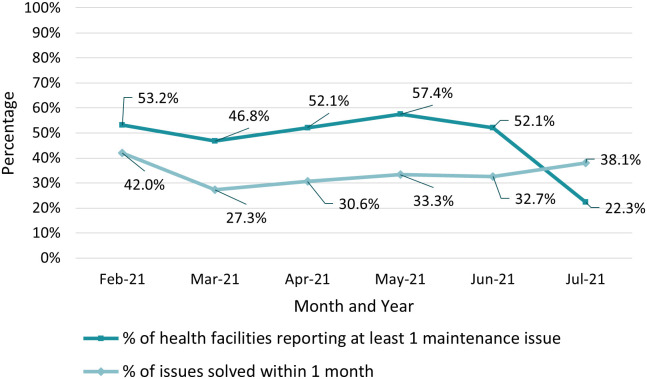
Proportion of health facilities reporting maintenance issues and proportion of issues solved within 1 month.

Over the evaluation period, 267 issues related to digital submission of data were reported by HFs. Issues were experienced by 74.5% (70/94) of the HFs at some point during the evaluation period, with no specific patterns by districts, HFs, or month. More than one-third (36.3% [97/267]) of the issues reported were problems with accessing the app or internet connections. Difficulties accessing the app were due to broken tablets (37.1% [36/97]) and faulty log-in credentials (23.7% [23/97]). Connectivity problems were mainly due to insufficient credit (5.2% [5/97]) or network quality (19.6% [19/97]) (Figure 4). These issues were experienced at similar rates in urban and rural facilities. Seven HFs reported a month in which a new malaria focal point still needed to be trained on the new system before being able to use the platform (7.2% [7/97]). Over a third (33.7% [90/267]) of issues were resolved within 1 month. Figure 5 details a time line of the proportion of HFs reporting maintenance issues and the proportion of issues solved within 1 month.

### Acceptability of the iMISS among target users.

Overall, malaria focal points at HF and district levels showed high acceptability toward the new system. They showed a good understanding of the purpose and content of the iMISS and appreciated the potential of the electronic submission of monthly reports to save commute times of HF staff traveling to DHOs to submit paper-based reports. One user pointed out that “an advantage [of using the] iMISS is that we can easily enter the data anywhere and anytime, and [it] allows us to visualize the data [and] it makes it easier for us to send the data already entered to colleagues at [the] district level without having to move….” Another user mentioned that the overall goal of the iMISS was to increase data-informed decision-making: “From the results, we will […] be able to plan according to the problems…. It also aims to see the quality of the information we have, the quality of the system itself, the quality of the program. This makes it easier for us to have the accurate and real planning….”

However, users also highlighted that entering data and using the tablet are time-consuming. They reiterated the issues reported through the quantitative component—synchronization issues, insufficient phone credit, and difficulties logging into the system: “…the only thing I see as time-consuming is the updating of the data in iMISS, because sometimes, we already have the data in SISMA (the monthly summary of malaria activities), and…in iMISS do not know what time it takes to aggregate the data, it is not fast, [and] it takes synchronization to visualize. I think the synchronization should be immediate….”

Although users reported high acceptance of the new system, they also reported irregular use of the iMISS for data-informed decision-making. Irregular discussion of the data on the platform was reportedly due to tight schedules and other, more important immediate issues: “…regarding the frequency, we aim to discuss monthly; [however,] some colleagues—because they have positions of leadership—have a tight schedule, and sometimes we have to look for time to discuss.”

## DISCUSSION

An integrated malaria information system for surveillance is essential for malaria control and elimination.^7^ An ideal integrated system incorporates all malaria-related data—such as information from the routine health information system, census or health survey information, central data storage and management—produces automated as well as customized outputs and allows feedback that leads to timely and targeted responses.^8^^,^^9^ Mozambique rolled out such a system, the DHIS2-based iMISS, to all districts in February 2021. This evaluation focused on assessing the data quality, adoption, maintenance, and acceptability of the iMISS, including electronic submission of monthly malaria reports in seven districts where the iMISS was extended to the HF level. For the first time, malaria staff at the HF level were able to view malaria-related information on the iMISS, as well as electronically submit surveillance information through tablet-based apps. Previous experiences from integrating malaria information systems—and allowing direct data entry at the HF level—stressed the importance of a transition phase to ensure migration with minimal disruption and to ensure “sufficient flexibility to account for further additions or changes identified during the implementation phase.”^10^

Overall, electronic submission of monthly malaria reports at the HF level was well accepted by key users 6 months after rollout. Despite more than 70% of health facilities experiencing occasional issues with system access and internet connectivity during the evaluation period, the impact on acceptability was minimal, as most issues were minor and temporary in nature.

Although reporting rates and timeliness of reporting were good (> 99%) and the iMISS reflected information from paper-based reports well, improvements are still required as only 86.1% of electronically submitted malaria cases on the iMISS were within 10% of cases reported through paper-based reports. More than 20% of HFs showed inaccurate malaria cases on the iMISS compared with paper-based reports at some point during the 6-month evaluation period. These data fidelity issues should be addressed at HF-level through prioritization of ongoing routine data quality audits. The DHOs should continue low-level performance analysis and targeted supervision visits at the HF level beyond quarterly visits until maturity of the system has been reached. The importance of regular on-site supervision visits to ensure the quality of the malaria surveillance system has been stressed in WHO guidelines and other surveillance system evaluations.^7^^,^^11^ Discrepancies were noted between the iMISS and the SISMA, which partially explains the low fidelity between the number of malaria cases reported on the iMISS compared with monthly paper-based reports. Postsubmission corrections are not reflected on the iMISS immediately, as each month only the current month’s data are retrieved, potentially leading to the observed discrepancies between iMISS and SISMA data. A new strategy should be implemented where postsubmission corrections are reflected on the iMISS in a timely manner and consistency between the two system is ensured.

Owing to remote data collection to measure the adoption of the iMISS for data-informed decision-making, a proxy indicator assessing the proportion of meetings where information from the iMISS was discussed was used. Although this indicator only measured system adoption by proxy, qualitative information substantiated that even though key users at district and HF levels showed a good understanding of the content and purpose of the new system, data were not routinely used for decision-making. This is not surprising, as the new platform still needs maturity; however, suboptimal utilization has been previously reported as a common problem with other surveillance systems, which report that “data that are collected but not used are an untapped resource.”^11^^,^^12^ Experiences from other countries show that effective strategic decision–making behavior is not solely changed by the presence of an integrated information system and an appropriate technical solution^13^^,^^14^ and that “building a culture of information use is not simply a question of technology.^15^” It is equally about political commitment^14^ and establishing organizational practices.^15^ A multilevel analysis from Ethiopia identified the lack of supervision and feedback mechanisms and lack of capacity at senior management level as reasons for low use of routine data.^15^ This was also identified in Guinea when their integrated health information system was rolled out.^16^^,^^17^ To improve data use, the format of decision-making meetings should be assessed, key users having pointed out that regular meetings where information could be reviewed were often deprioritized. In addition, feedback from malaria focal points should be considered for developing user-friendly, customized outputs that are simple to interpret. Furthermore, the NMCP should prioritize the development of clear guidelines, responsibilities, and training on data-to-action meetings for lower- and senior-level management. The establishment of province iMISS focal points is advisable for facilitating timely solutions to maintenance issues, and the establishment of a functioning central coordinating body is essential for maintaining the performance of the platform and political commitment. Key partners including the NMCP, key users at all levels, developers, and implementing partners should regularly share information to resolve technical issues in a timely way and make the iMISS a user-friendly platform to facilitate trust in the system and encourage uptake. To align expectations and coordinate iMISS adoption, the NMCP created an iMISS task force in 2021 to liaise with provincial and district focal points. This was aimed at streamlining data flow expectations, documenting uptake challenges, and rapidly addressing any system issues.

## CONCLUSION

Integrated malaria information systems are essential for malaria control and elimination. Overall, the iMISS and electronic submission of monthly reports at the HF level in Mozambique are effective in achieving high-level data quality and acceptability among key users. Continued political commitment and more timely execution of issue management work flows are crucial to ensure trust in the new platform and facilitate higher levels of data use. The lessons learned from the rollout of the iMISS to HFs in seven districts will be used to guide the optimization of the nationwide rollout to the HF level.

## Data Availability

The data that support the findings of this study are available from the corresponding author upon reasonable request.

## References

[1] World Health Organization, 2021. World Malaria Report 2021. Geneva, Switzerland: WHO.

[2] Malaria Consortium, 2018. Technical Brief: Malaria Surveillance System Performance in Mozambique: A Comprehensive Assessment. Maputo, Mozambique: Malaria Consortium.

[3] Republic of Mozambique, Ministry of Health, 2017. National Malaria Control Programme: Malaria Strategic Plan 2017–2022. Maputo, Mozambique: Ministry of Health Mozambique.

[4] Malaria Consortium, 2019. Project Brief: Strengthening Malaria Surveillance for Data-Driven Decision Making in Mozambique. Maputo, Mozambique: Malaria Consortium.

[5] Clinton Health Access Initiative, 2019. User Requirements Document – iMISS Integrated Malaria Information and Storage System.

[6] Manya A Braa J Øverland L Titlestad O Mumo J Nzioka C, 2012. *National roll out of District Health Information Software (DHIS 2) in Kenya, 2011 – central server and cloud based infrastructure*. IST-Africa 2012 Conference, May 9–11, 2012, Dar es Salaam, Tanzania.

[7] World Health Organization, 2018. Malaria Surveillance, Monitoring and Evaluation: A Reference Manual. Geneva, Switzerland: WHO.

[8] Ohrt C Roberts KW Sturrock HJW Wegbreit J Lee BY Gosling RD, 2015. Information systems to support surveillance for malaria elimination. Am J Trop Med Hyg 93: 145–152. 26013378 10.4269/ajtmh.14-0257 PMC4497887

[9] Aide P , 2019. Setting the scene and generating evidence for malaria elimination in southern Mozambique. Malar J 18: 190. 31170984 10.1186/s12936-019-2832-9 PMC6554892

[10] Fu C Lopes S Mellor S Aryal S Sovannaroth S Roca-Feltrer A, 2017. Experiences from developing and upgrading a web-based surveillance system for malaria elimination in Cambodia. JMIR Public Health Surveill 3: e30. 28615155 10.2196/publichealth.6942 PMC5489705

[11] Wangdi K Sarma H Leaburi J McBryde E Clements ACA, 2020. Evaluation of the malaria reporting system supported by the District Health Information System 2 in Solomon Islands. Malar J 19: 372. 33069245 10.1186/s12936-020-03442-y PMC7568381

[12] West N Gyeltshen S Dukpa S Khoshnood K Tashi S Durante A Parikh S, 2016. An evaluation of the national malaria surveillance system of Bhutan, 2006–2012 as it approaches the goal of malaria elimination. Front Public Health 4: 167. 27595095 10.3389/fpubh.2016.00167 PMC4990597

[13] Mutemwa RI, 2006. HMIS and decision-making in Zambia: re-thinking information solutions for district health management in decentralized health systems. Health Policy Plan 21: 40–52. 16319088 10.1093/heapol/czj003

[14] Sæbø JI Kossi EK Titlestad OH Tohouri RR Braa J, 2011. Comparing strategies to integrate health information systems following a data warehouse approach in four countries. Inf Technol Dev 17: 42–60.

[15] Chikumba PA Ramussen SL, 2016. *Management and use of health information in Malawi and Burkina Faso: the role of technology*. 2016 IST-Africa Week Conference, May 11–13, 2016, Durban, South Africa.

[16] Chanyalew MA Yitayal M Atnafu A Tilahun B, 2021. Routine health information system utilization for evidence-based decision making in Amhara national regional state, northwest Ethiopia: a multi-level analysis. BMC Med Inform Decis Mak 21: 28. 33499838 10.1186/s12911-021-01400-5 PMC7836202

[17] Reynolds E , 2021. Implementation of DHIS2 for disease surveillance in Guinea: 2015–2020. Front Public Health 9: 761196. 35127614 10.3389/fpubh.2021.761196 PMC8811041

